# New Developments in Diagnosis and Management of Acquired Hemophilia and Acquired von Willebrand Syndrome

**DOI:** 10.1097/HS9.0000000000000586

**Published:** 2021-06-01

**Authors:** Frank W. G. Leebeek

**Affiliations:** Department of Hematology, Erasmus MC, University Medical Center Rotterdam, Rotterdam, the Netherlands

## Abstract

Acquired hemophilia A and acquired von Willebrand syndrome are rare, but life-threatening bleeding disorders that require prompt diagnosis and treatment by hematologists. Acquired hemophilia A is defined as an acquired severe bleeding tendency caused by autoantibody formation against coagulation factor VIII. Acquired von Willebrand syndrome is characterized by a new onset bleeding tendency caused by a reduced concentration and/or function of von Willebrand factor. These disorders are associated with a variety of underlying disorders, including various hematological malignancies, for example, plasma cell disorders, lymphoproliferative disorders, monoclonal gammopathy of undetermined significance, and myeloproliferative neoplasms. It is of utmost important to recognize these acquired bleeding disorders in these patients who are at risk for severe bleeding, and to perform additional diagnostic hemostasis laboratory evaluation. This will enable immediate diagnosis of the acquired bleeding disorder and management of both the bleeding episodes and the causative underlying disorder. In recent years, several new etiological factors for acquired hemophilia A, such as treatment with immune checkpoint inhibitors or DPP-4 inhibitors and SARS-CoV2 infection, and for acquired von Willebrand syndrome, for example, left ventricular assist devices, have been identified and also new treatment options have become available. In this concise review, the most recent data on etiology, diagnosis, and treatment of acquired bleeding disorders are presented and discussed.

## Introduction

Acquired hemophilia A (AHA) and acquired von Willebrand syndrome (aVWS) are rare, but life-threatening bleeding disorders that require prompt diagnosis and treatment by hematologists. Acquired hemophilia is defined as an acquired severe bleeding tendency in a patient with a negative bleeding history and a negative family history of bleeding caused by autoantibody formation against a coagulation factor.^1^ It mainly concerns antibodies directed against coagulation factor VIII (FVIII) (>90% of cases), but may also be directed toward other coagulation factors including FVII and FV. AHA can occur in association with autoimmune disorders and (hematological) malignancies, but in 50%–60% of cases, no etiological factor is found.^2^ aVWS is characterized by an acquired severe bleeding tendency in a patient with a negative bleeding history and a negative family history of bleeding caused by a reduced concentration or function of von Willebrand factor (vWF).^3^ aVWS can have many different etiologies, varying from hematological malignancies, immunological disorders, drugs, to cardiovascular causes.^4^ Because of these various causes underlying aVWS, the management of this disorder is highly variable. Because both disorders are rare and the diagnostic work-up and optimal treatment very individually determined, the management of these disorders remains challenging, not only in treating bleeding complications, but also for treatment of the underlying disorder.

## Acquired hemophilia

### Case 1

A 70-year-old male, without any significant medical history or prior bleeding diathesis, presented with hematuria, for which he was referred to the urologist. Extensive investigations did not reveal a cause of bleeding. In the following weeks, he developed spontaneous large cutaneous hematomas on the arms, left leg, and a painful calf. He was referred to the hemophilia treatment center for further investigation to reveal the cause of bleeding. Physical examination showed multiple large hematomas and a swollen painful left calf muscle, suspected for a muscle bleed. In addition, several enlarged inguinal lymph nodes were noted. Laboratory results showed an activated partial thromboplastin time (APTT) of 70 seconds (normal: 22–32 s) and a normal prothrombin time. A mixing experiment with 1:1 patient:normal plasma revealed no normalization of the APTT (53 s). Determination of individual intrinsic coagulation factors showed a FVIII:C activity of <0.01 U/mL. vWF antigen and activity were normal. A titer of anti-FVIII antibodies was 71 BU. This confirmed the diagnosis of AHA. He was successfully treated with factor eight inhibitor bypassing activity 50 U/kg twice daily for hematuria and the muscle bleed. Immunosuppressive treatment was started with prednisone in a dose of 1 mg/kg. Additional investigations revealed increased immunoglobulin M (IgM) (2.95 g/L [normal 0.45–2.30 g/L]) and an IgM monoclonal protein, which was not quantifiable. Peripheral blood showed a monoclonal B-cell population, with an immunophenotype suggestive of lymphoplasmacytic lymphoma (LPL). CT scan revealed generalized lymphadenopathy. A lymph node biopsy was not performed because of the severe bleeding tendency. He was treated with 8 courses of R-CVP (rituximab, cyclophosphamide, vincristine, and prednisone). This resulted in a gradual decrease of the anti-FVIII titer, rise of FVIII, and amelioration of the bleeding phenotype. CT scan after 8 courses of chemotherapy revealed a complete remission. The patient has been followed for 4 years and LPL is still in remission, without a recurrence of AHA.

## Epidemiology and etiology

Acquired hemophilia is a rare bleeding disorder with an estimated incidence of 1–4 patients per million, although the incidence is increasing.^2,5^ It most frequently occurs in elderly individuals, as in our patient, with a peak incidence between the age of 70 and 80 years. In several registries, the median age of patients was 73 years. However, AHA can also occur at a younger age, even in children, depending upon the underlying cause.^6^ AHA also occurs in young women, in whom AHA is pregnancy-related (5% of AHA cases). In Table 1, the main underlying disorders associated with AHA are listed. Around 20% of AHA cases are associated with malignancies, of which around one-third hematological malignancies, including lymphoma, chronic lymphatic leukemia, plasma cell disorders, and monoclonal gammopathy of undetermined significance (MGUS).^7^ The second most common causes are autoimmune disorders like systemic lupus erythematosus, rheumatoid arthritis, and immune-mediated dermatological diseases.^2,8,9^ Viral infections, such as HIV and influenza, are rare causes of AHA. Recently, 2 AHA cases associated with SARS-CoV-2 were reported.^10,11^ Certain drugs, for example, antibiotics, may lead to AHA. Several cases of AHA related to newly developed and widely used drugs have recently been reported. Treatment with alemtuzumab was associated with AHA in multiple sclerosis patients.^12–14^ AHA has also been reported associated with the use of immune checkpoint inhibitors, including pembrolizumab, and during DDP-4 inhibitor use in patients with diabetes.^15,16^ In around 50%–60% of AHA cases, no cause or underlying disease is recognized.^2,8,9^

**Table 1.** Etiology and Underlying Disorders of AHA and aVWS (Based on Registries^2,8,9,19,20^)

	AHA		aVWS	
Etiology Type	Underlying Disorder	Frequency (%)	Underlying Disorder	Frequency (%)
Hematological malignancies	Lymphoma	5%–10%	Plasma cell disorder	48%
	CLL		MGUS (IgG, IgM)	
	Plasma cell disorders		CLL	
	MGUS		Essential thrombocythemia, with very high platelet counts	
Solid malignancies	Prostate carcinoma, lung carcinoma, colon carcinoma, gastric cancer	12%	Urothelial cell carcinoma	Rare
Immunological disorder	Autoimmune disorders (SLE, rheumatic disease)	9%–20%	Autoimmune disorders (SLE)	<5%
Drug-induced	Antibiotics	2%–5%	Valproic acid, cephalosporin	Rare
	Alemtuzimab			
	Pembrolizumab			
	DDP-4 inhibitors			
Cardiovascular	n.a.		Aortic stenosis	20%
			Congenital heart disease	
			Left ventricular assist devices	
			ECMO-induced	
Dermatological disorders	Parapemphigus	1%–4%	n.a.	n.a.
Pregnancy		2%–15%	n.a.	n.a.
Infections	HIV, influenza, SARS-Cov2	2%–4%	Viral infections rarely reported	Rare
Idiopathic/unknown cause		44%–67%		<5%
Other various causes		12%–17%	Hyperfibrinolysis (plasmin-mediated), glycogen storage disease, uremia, hypothyroidism	<10%

AHA = acquired hemophilia A; aVWS = acquired von Willebrand syndrome; CLL = chronic lymphatic leukemia; ECMO = extracorporal membrane oxygenation; MGUS = monoclonal gammopathy of undetermined significance; n.a. = not associated; SLE = systemic lupuis erythematosus.

## Bleeding symptoms

Most AHA patients present with large to very large subcutaneous hematomas and muscle bleeding. These muscle bleeds occur in the lower and upper extremity and also retroperitoneal. Patients may also suffer from gastrointestinal bleeding and hematuria.^17^ The bleeding pattern in AHA patients is strikingly different from patients with inherited forms of hemophilia, who mainly suffer from joint bleeds and only present with (mild) cutaneous hematomas after trauma. The large cutaneous hematomas should especially trigger physicians to immediately consider AHA. Postoperative bleeding may occur in patients in whom the diagnosis was not evident before the intervention. Several registry studies reported up to 10% of AHA patients without bleeding symptoms, but in whom the diagnosis was made because of a prolonged APTT.^2^ In elderly patients who are treated with anticoagulant drugs, the diagnosis of AHA is sometimes delayed due to the fact that the bleeding is attributed to the use of anticoagulants.^18^ However, the bleeds observed during anticoagulant use, even in the case of overdosing or high international normalized ratio, do not resemble those of AHA. Because AHA is such a rare disease, the time between the first bleeding manifestation and diagnosis is long, as was also found in a recent Dutch study in 143 AHA patients. The diagnostic delay was 22 days (median, IQR 8–77).^9^ This is partly due to patient delay but is also caused by doctor delay, which was 2 days (median, IQR 0–10) after first medical evaluation. Several retrospective studies have investigated the association between FVIII levels at diagnosis and bleeding risk, and found that FVIII levels are not very predictive for bleeding.^17,19,20^ The risk of bleeding complications declines with increasing FVIII levels after start of immunosuppressive treatment.^17^ Despite the major bleeding complications in AHA, fatal bleeding occurs in a minority of patients. In a large German study (GTH-AH 01/2010 study) on AHA in 102 patients, the rate of fatal bleeding was 3%, and in the Dutch study 7.7%.^9,17^

## Laboratory diagnosis of AHA

Recently, international recommendations on the diagnosis and management of acquired hemophilia were published.^21^ The recommendations in the guideline are mainly based on registry data and clinical experience, and not on controlled clinical trials due to the rare occurrence of acquired hemophilia. If a patient presents with new onset severe bleeding tendency, a screening panel consisting of APTT and prothrombin time (PT) should be performed. In the case of an isolated APTT prolongation, a mixing study with 1:1 patient plasma:normal plasma should be performed. In case the mixing study still shows a prolonged APTT, an inhibitor is suspected and levels of intrinsic coagulation factors should be measured.^21^ Since over 90% of acquired hemophilia is caused by antibodies against FVIII, this should be determined first. In case of reduced factor FVIII, AHA is highly likely and the antibody titer should be determined using the Nijmegen modification of the Bethesda assay.^22^ In contrast to antibodies occurring in patients with inherited hemophilia that follow type 1 kinetics, the kinetics of the FVIII antibodies in AHA may be different, that is, type 2 kinetics, which may influence the measurement of the titer of the AHA FVIII antibody. It is important to quantify the titer at diagnosis because management and prognosis of AHA may depend on the titer.^8^

## Management of AHA

Because of the severity and rarity of AHA, patients with AHA should be managed in a specialized hemophilia treatment center as soon as the diagnosis is either suspected or confirmed.^21^ We have had several patients over the years for whom a delay occurred between diagnosis and treatment because they were not referred immediately. In some AHA-suspected patients, this also led to severe bleeding complications because they were exposed to high bleeding risk interventions, such as biopsies or placement of intravenous central lines, which should be avoided in these patients.

## Management of bleeding with hemostatic agents

AHA patients presenting with severe bleeding complications should be treated with FVIII bypassing agents, such as activated prothrombin complex concentrate (aPCC) or recombinant activated FVII (rFVIIa).^21,23^ Both of these drugs have been used successfully for many decades, and bleeding cessation is achieved in >90% of cases.^17^ aPCC can be given 2–3 times daily at 50–100 U/kg body weight, with a maximum of 200 U/kg per 24 hours (Table 2). rFVIIa is administered intravenously in a dose of 90 µg/kg followed by a similar dose every 2–3 hours until hemostasis is achieved. The duration of treatment with aPCC and rFVIIa depends on the type and severity of bleeding. In the case of failure of one of these agents, one might successfully switch to the other product.^21^ In case bleeding persists, recombinant porcine FVIII (rpFVIII) can be used. rpFVIII has been registered for the indication of AHA for several years. According to the label, the start dose is 200 U/kg with the aim to achieve a level of FVIII of 100%, and following doses should be targeted at a trough FVIII level >50%. A recent study in 18 patients showed that lower doses than the FDA-approved dose can also be used. In patients in whom rpFVIII was used upfront, an initial dose of 100 U/kg was used, followed by doses after 4–8 hours depending on the reached FVIII level.^24^ It is recommended to closely monitor FVIII levels over time and to keep the levels of FVIII >50% in patients with severe bleeding.^21^ In a minority of patients, the autoantibodies against human FVIII are cross-reactive to the administered recombinant porcine FVIII. This may reduce the efficacy and the recovery of rpFVIII.^25^ In the case of lack of availability of one of the bypassing agents, recombinant human FVIII (rhFVIII) can be used. However, in most cases, rhFVIII is not useful because it either does not result in the increment of FVIII, or achieved FVIII levels will decrease rapidly due to fast FVIII clearance. Desmopressin (DDAVP) is not recommended in patients with AHA.^21^ Emicizumab is a bispecific FVIII-mimetic antibody that is registered for use in patients with inherited hemophilia either with or without inhibitory antibodies, but not for AHA. Recently, Knoebl et al^26^ reported successful therapeutic use of emicizumab in a case series of 12 patients with AHA. A dose of 3 mg/kg subcutaneously once per week for 2–3 doses, followed by 1.5 mg/kg every three weeks, resulted in normalization of the APTT and cessation of bleeding. Bypassing agents could be stopped after 1.5 days. Several other cases using other dose regimens of emicizumab in AHA have been reported and are reviewed by Tiede et al.^27^ It was concluded that emicizumab may be able to shorten the duration of initial bleeds and recurrent bleeding, but more data from prospective clinical trials is needed.

**Table 2.** Treatment of Bleeding in AHA

Treatment for AHA	Dose	Monitoring	Remarks
aPCC (FEIBA)	50–100 U/kg every 8–12 h (max 200 U/kg/d)	No laboratory monitoring possible	Continue till hemostasis is achieved
Recombinant FVIIa (NovoSeven)	90 µg/kg every 2–3 h	No laboratory monitoring possible	Continue till hemostasis is achieved
Recombinant porcine FVIII (Obizur)	200 U/kg intravenously initial dose, followed based on FVIII levels achieved. Trough FVIII levels of >50% in the case of severe bleeding	Closely monitor FVIII levels because of potential cross-reactivity of antiFVIII antibodies to porcine FVIII, although the titers do not correlate well with the response to rpFVIII. Dosing based on trough target levels of 50-70% for severe life-threatening bleeds and 30-50% for other bleeds	Lower initial doses may be used (100 U/kg) followed immediately with recovery level assessment and tailoring treatment base on trough levels
FVIII concentrate	50–100 U/kg, depending upon FVIII levels achieved intravenously	Monitoring of recovery and trough FVIII levels	In most patients. no increment is found, but may depend upon inhibitor titer. Use in case no bypassing agents are available
DDAVP	0.3 µg/kg every 16–24 h intravenously	Monitor FVIII levels post DDAVP	Not recommended
Emicizumab (Hemlibra)	3 mg/kg initial dose once weekly for 3 wks, followed by 1.5 mg/kg every 3 wks subcutaneous; In the largest case series a median of 5 doses (IQR 3–7) were administered	No target concentration of emicizumab known	NOT registered for this indication; data based on several case studies
		Measure hFVIII with chromogenic assay	

AHA = acquired hemophilia A; aPPC = activated prothrombin complex concentrate; DDAVP = desmopressin; FVIII = coagulation factor VIII; FEIBA = factor eight inhibitor bypassing activity.

## Management of underlying cause

The second aim of treatment AHA is the eradication of the auto-antibodies against FVIII. Irrespective of the underlying cause, treatment with immunosuppressive agents should be started immediately after diagnosis. Initial treatment consists of corticosteroids (prednisone 1 mg/kg for 4–6 wks) with or without the use of cyclophosphamide.^21^ Despite the lack of evidence from randomized studies in AHA, several guidelines suggest to use a more intensive immunosuppressive regimen in case of very low FVIII levels (<1%) and a high antibody titer (>20 BU). In these patients, it is recommended to additionally use cyclophosphamide (2 mg/kg per day for a maximum of 6 wks) or rituximab (375 mg/m^2^ once weekly for 4 wks). This recommendation is based on the finding that combined immunosuppressive treatment resulted in shortening the time to remission, although this was not observed in all studies.^9,28^ Instead of using cyclophosphamide, mycophenolate mofetil (dose of 1 g/d for 1 wk, followed by 2 g/d) may also be used.^21^ High-dose intravenous immunoglobulins (IVIGs) are not recommended. For patients with failed response to immunosuppression, immunoadsorption can be used to remove the auto-antibodies to FVIII.^29^ Treatment of the underlying cause—for instance in our patient, treating LPL with chemoimmunotherapy—or stopping a drug associated with AHA, may also lead to cure of AHA and reduce the risk of recurrence.

## Prognosis of AHA

Although complete remission is reached in around 50% of patients receiving first-line immunosuppressive treatment, most patients (80%) eventually reach complete remission of the AHA after the first presentation and (combined) immunosuppressive treatment, after a median time period of 10 weeks.^8,9^ After stopping immunosuppression, around 25% of patients relapse. In a recent review on pregnancy-associated AHA, the risk of relapse was 22% in subsequent pregnancies.^30^ AHA is a severe bleeding disorder and may lead to mortality in a considerable number of patients. In a German study, mortality after 1 year was 32%, which is in line with Dutch data that showed overall mortality of 38% after long-term follow-up.^8,9^ Mortality is mainly due to either infectious complications related to immunosuppressive treatment (around 20% of deaths) or the underlying disease (malignancy).

## Acquired von Willebrand Syndrome

### Case 2

**Figure 1.**
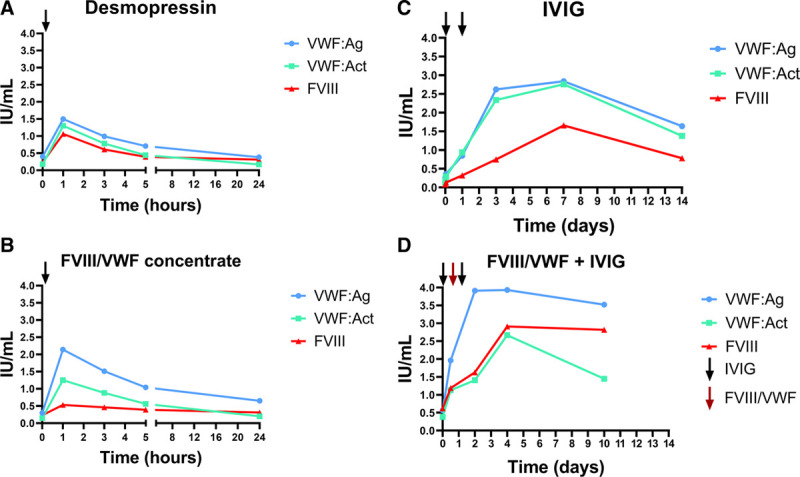
**The responses to various hemostatic treatments administered to patient 2 with aVWS are depicted.** (A) DDAVP (0.3 µg/kg IV); (B) FVIII/VWF concentrate (50 FVIII U/kg [=120 VWF:RCo U/kg] IV); (C) IVIGs 1 g/kg on day 0 and 1; (D) combination of IVIG 1 g/kg on day 0 and 1 followed by FVIII/VWF concentrate on day 0 (50 FVIII U/kg [=120 VWF:RCo U/kg] IV). aVWS = acquired von Willebrand Syndrome; DDAVP = desmopressin; FVIII = coagulation factor VIII; IVIG = intravenous immunoglobulin.

A 34-year-old male who had undergone several dental and surgical interventions without bleeding in the past presented with recurrent hematuria and hematospermia, for which no local cause was found by the urologist. At referral to the hematologist, his additional bleeding history revealed nose bleeds and hematomas after minor trauma. His identical twin brother had no bleeding history. Physical examination at referral was normal. Laboratory investigations revealed a platelet function analyzer closure time of >300 seconds (normal <150), prolonged APTT (53 s), with normalization in the mixing study, FVIII:C 0.02 IU/mL, VWF:Antigen 0.18 IU/mL, and VWF:RCo <0.12 IU/mL. The multimer pattern was abnormal, lacking high molecular weight multimers. Von Willebrand disease was diagnosed, but an aVWS was more likely based on the new onset bleeding tendency and the negative family history. Additional examination showed a monoclonal IgG-lambda protein, which was not quantifiable, classified as IgG MGUS. Total IgG was 13.9 g/L (normal 7.0–16 g/L) and IgA and IgM were also normal. A FVIII/VWF concentrate test dose was administered of 50 U/kg FVIII (=120 U/kg VWF RCo). This resulted in a short lasting normalization of VWF:Ag (estimated half-life of 6 h) (Figure 1). Intravenous DDAVP administration also resulted in a short-term rise of FVIII and VWF. Infusion of IVIGs (1 g/kg day 0 and 1) resulted in a normalization of FVIII and VWF within 2 days which lasted for at least 2 weeks (Figure 1). Administration of IVIG immediately followed by 1 dose of FVIII/VWF concentrate (50 U FVIII/kg) resulted in instantaneous normalization of VWF and FVIII, and this regimen was used for several severe bleeding episodes. Remarkably, FVIII:C was lower than the VWF:Ag level, and the FVIII increase was lower than expected after administering FVIII/VWF concentrate, which appeared to be due to a lupus-like anticoagulant activity, with a prolonged APTT lupus and increasing levels of FVIII in serial dilution studies. No inhibitor against FVIII was detected by the Bethesda assay. Three years later, the patient also developed a monoclonal protein IgM-kappa and abdominal lymphadenopathy, for which he received rituximab (IV 375 mg/m^2^ for 4 wks), with resolution of lymphadenopathy and reduction of IgM. The quantity of the IgG-lambda monoclonal protein did not change and aVWS persisted. He has since been treated only in the case of bleeding, including several muscle and joint bleeds, with IVIG and FVIII/VWF concentrate. In the case of elective interventions, he is treated with IVIG alone 2 days before the surgery without additional factor concentrate. No other treatment for the MGUS has been given.

## Epidemiology and etiology

aVWS is a rare bleeding disorder, which is nearly always associated with an identifiable underlying cause. About half of the patients have a lymphoproliferative disorder, 20% a cardiovascular cause, including aortic stenosis and left ventricular assist device (LVAD), 15% a myeloproliferative neoplasm (mainly essential thrombocythemia), and several less frequently occurring causes^31^ (see Table 1). For a more extensive list of pathogenic causes, see a review of Federici et al.^4^ The mechanisms by which these disorders cause aVWS include specific antibodies or nonspecific autoantibodies that form circulating immune complexes and lead to enhanced clearance of VWF, adsorption of VWF on malignant cells, or enhanced shear stress, resulting in excessive cleavage of VWF by ADAMTS13 after unfolding of VWF due to shear stress.^4^ In patients with lymphoproliferative disorders including plasma cell dyscrasias, various mechanisms may lead to aVWS, but in none of these patients autoantibodies against VWF are found.^32^ In a minority of patients, aVWS is caused by decreased synthesis of VWF (eg, hypothyroidism), plasmin-mediated VWF cleavage associated with hyperfibrinolysis, or an unknown mechanism (glycogen storage disease; drugs).^33–35^ Due to the increased implantation of permanent LVADs and use of extracorporal membrane oxygenation, the prevalence of aVWS is increasing. Cardiovascular causes account for up to 40% of the currently diagnosed aVWS, as discussed in recent reviews.^31,36^ LVAD-associated aVWS is seen mainly with first- and second-generation devices, and is seen in a lesser extent in pulsatile third generation LVAD, such as Heartmate III.^37,38^

## Bleeding symptoms

aVWS results in a severe bleeding diathesis, not only due to the reduced VWF level, but also due to the concomitant decrease of FVIII. Therefore, the bleeding spectrum ranges from mucosal bleeding, including nose bleeds, cutaneous hematomas, hematuria, and gastrointestinal bleeding, to joint and muscle bleeds.^4^ The association between aVWS and gastrointestinal bleeding is well known in Heyde syndrome, typically characterized by aortic stenosis, aVWS, and angiodysplasia.^39^ aVWS as complication after LVAD implantation may also be associated with angiodysplasia leading to gastrointestinal bleeding in up to one-third of the patients.^40^ The risk of bleeding is especially increased in patients with reduced high molecular weight multimers.^38,41^ Recent studies have shown that VWF has antiangiogenic properties, and reduction of VWF levels, in particular of the high molecular weight multimers, may therefore be associated with angiodysplasia.^42^ This is not only observed in aVWS but is also frequently encountered in patients with inherited von Willebrand disease, especially in type 2A and type 3.^42–44^

## Laboratory diagnosis of aVWS

The diagnosis of aVWS is based on the history of the patient, lack of family history, and laboratory assessment of VWF parameters. Screening tests for primary and secondary hemostasis are abnormal. The closure time measured using platelet function analyzer 100/200 is prolonged, and the APTT is also prolonged. A 1:1 mixing study with normal plasma shows normalization of the APTT because in contrast to AHA, no inhibitor against FVIII is present. VWF:Antigen (VWF:Ag), VWF:ristocetin cofactor activity (VWF:RCo) or another comparable functional test (eg, VWF:GPIbM), and VWF:collagen binding activity (VWF:CB) should be determined.^4^ Additionally, VWF multimer composition should be assessed with agarose gel electrophoresis. FVIII:C activity is also reduced in most aVWS patients and should also be measured. In the case of LVAD associated aVWS, levels of FVIII:C, VWF:Ag and VWF:RCo may be within the normal range or even above.^38^ The aVWS diagnosis is based on the abnormal multimer pattern, lacking high molecular weight multimers, and either reduced VWF:RCo/VWF:Ag ratio or VWF:CB/VWF:Ag ratio. Therefore, in these patients, the VWF:RCo/VWF:Ag ratio should be used to diagnose aVWS instead of VWF:Ag levels or VWF:RCo activity.^36,38^

## Management of aVWS

### Management of bleeding in aVWS with hemostatic agents

The management of bleeding episodes is strongly dependent upon the cause of aVWS. In patients with IgG MGUS, as in our patient, treatment of (severe) bleeding should consist of IVIG in combination with FVIII/VWF concentrate, as was shown in our case.^4^ In patients with IgM-MGUS-associated aVWS, IVIG is not useful and treatment may consist of factor concentrates to stop bleeding and plasmapheresis to reduce the IgM M-protein.^32^ In patients with cardiovascular causes, FVIII/VWF concentrate can be administered, although the half-life of the infused VWF may be shortened due to increased proteolysis of the exogenous VWF.^45,46^ In some cases, for instance in the case of mild bleeding episodes, DDAVP; in a dose of 0.3 µg/kg intravenously can be used.^4^ Gastrointestinal bleeding in aVWS patients may be hard to treat and reoccur frequently, despite endoscopic interventions. This can be treated with FVIII/VWF concentrate with the addition of fibrinolysis inhibitors (eg, tranexamic acid 3–4 g/daily).^47,48^ In the case of failure, rFVIIa has been administered to treat severe bleeding episodes. The regular dose is 90 µg/kg IV, followed by repeated dosing until cessation of bleeding.^31^ Incidental cases have been reported on the use of antiangiogenic drugs to treat angiodysplasia-associated gastrointestinal bleeding, including thalidomide or statins; however, this was not always successful.^49^ Potential novel treatments for LVAD-associated aVWS are in development based on the inhibition of ADAMTS13, for instance by administration of ADAMTS13-inhibiting monoclonal antibodies.^50^

### Management of underlying cause of aVWS

The treatment of the underlying cause of aVWS is variable and based on the nature of the causative disorders. Unfortunately, a frequently occurring cause, IgG-MGUS is hard to treat. Immunotherapy or chemotherapy is not successful in most MGUS cases because the monoclonal protein will persist in very low concentrations.^51^ Due to the high avidity of the monoclonal protein toward VWF, resolution of aVWS will not be achieved, although an incidental case of bortezomib induced remission has been reported.^32,52^ In patients with cardiovascular-associated aVWS, the treatment depends on the underlying cause, which also determines the ability to cure aVWS. Explantation of the LVAD leads to immediate resolution of aVWS, and (transcatheter) aortic valve replacement may resolve aortic stenosis-related aVWS.^53,54^

## Prognosis of aVWS

Because many of the underlying causes of aVWS are difficult to treat, many patients will suffer long-term—if not lifelong—from aVWS. In our patient with an underlying IgG MGUS, treatment with immunosuppressive agents (rituximab) was not successful. The prognosis may be favorable with other causes of aVWS, for instance in aVWS patients with hypothyroidism, in whom treatment with thyroxine results in normalization of VWF.^33^ Also, in patients with myeloproliferative neoplasm who present with aVWS due to high platelet counts, cytoreductive therapy will normalize VWF levels and function.^55,56^ The mortality of patients with aVWS is mostly dependent upon the underlying cause, and no data on fatality rates due to bleeding is available.

## Differences and similarities between AHA and aVWS

As is indicated in the above paragraphs, AHA and aVWS present with a severe bleeding phenotype and even with similar bleeding symptoms. In our 2 patients, hematuria was the reason for referral to the hematologist. The typical bleeding pattern of both acquired bleeding disorders is different, however, and the pathogenic mechanisms also differ. Both disorders have many different etiologies, and the management of bleeding episodes is also dependent upon the underlying disorder. In Table 3, the differences and similarities between these 2 acquired bleeding disorders are summarized.

**Table 3.** Differences and Similarities Between AHA and aVWS

	AHA	aVWS
Incidence	1–4 per million	Very rare, exact incidence unknown
Etiology	Autoantibodies against FVIII	Complex and various underlying mechanisms leading to reduced VWF
		M-protein with affinity to VWF
		Proteolysis of VWF by ADAMTS13
		Adsorption of VWF by malignant cells
Underlying disorders	(Hematological) malignancy, MGUS, auto-immune disorders pregnancy, drugs 50% no underlying cause	Autoimmune disorders, MGUS (hematological) malignancy, cardiovascular causes, drugs
		Always associated with underlying cause
Age of presentation	Mainly elderly population	Depending upon underlying cause
Diagnosis	Prolongation APTT, also in mixing test, reduced FVIII, normal VWF parameters, autoantibody against FVIII measurable with Bethesda assay	Prolongation APTT. Normalization APTT in mixing test, prolonged closure time (PFA)
		Reduced VWF:Ag and VWF:RCo, Reduced FVIII
		VWF multimer pattern may be abnormal
		Reduced VWF:RCo/VWF:Ag ratio in LVAD associated aVWS
Main bleeding Symptoms	Large cutaneous hematomas	Mucosa-associated bleeding, gastrointestinal bleeding
	Muscle bleeds	Joint bleeds
Treatment of bleeding	aPCC, recombinant FVIIa, recombinant porcine FVIII	In the case of IgG MGUS: IVIG and FVIII/VWF concentrate
	(rFVIII concentrate)	In case of IgM MGUS: FVIII/VWF concentrate/plasmapheresisDDAVP
	Tranexamic acid (additional)	
	Emicizumab (case series; not registered for use in AHA)	
		Tranexamic acid (additional)
		Recombinant FVIIa (in the case of failure of other interventions)
		In case of cardiovascular cause: FVIII/VWF concentrate
Treatment of underlying disorder	Treatment with prednisone with or without additional immunosuppressive treatment (cyclophosphamide, rituximab, MMF)	Strongly dependent upon underlying disorder
	Immunoadsorption of anti-FVIII	
Prognosis	60% remission	Chronic disorder and prognosis strongly depending upon underlying cause
	30%–40% mortality	Mortality data not available

AHA = acquired hemophilia A; aPPC = activated prothrombin complex concentrate; APTT = activated partial thromboplastin time; aVWS = acquired von Willebrand syndrome; CLL = chronic lymphatic leukemia; DDAVP = desmopressin; FVIII = coagulation factor VIII; MGUS = monoclonal gammopathy of undetermined significance; MMF = mycophenolate mofetil; PFA = platelet function analyzer.

## Conclusions

The acquired bleeding disorders AHA and aVWS are rare, and difficult to diagnose and treat. Patients are at risk of severe, even potentially fatal bleeding, and require treatment in hemophilia treatment centers with expertise. The typical clinical presentation is a new onset bleeding tendency, without a family history, mainly occurring at advanced age. The very extensive cutaneous hematomas are highly suggestive of AHA, whereas the combination of mucosal bleeding, gastrointestinal bleeding, and muscle/joint bleeds may be more indicative of aVWS. The diagnosis should be based on extensive hemostatic evaluation, including screening tests for primary and secondary hemostasis, FVIII, and VWF measurement. Treatment of bleeding episodes and of an underlying disorder should immediately be started. Unfortunately, most recommendations on management of both bleeding disorders still depend on expert opinion and registry data. Therefore, prospective studies are needed to develop a more evidence-based approach.

## Acknowledgment

I would like to thank Calvin van Kwawegen for composing Figure 1.

## Disclosures

FWGL received unrestricted research grants from CSL Behring, Shire/Takeda, Sobi and uniQure. He is a consultant for CSL Behring, Shire/Takeda, Biomarin and uniQure, of which the fees go to the University. He received travel support from Sobi. He is a DSMB member of a study sponsored by Roche.
